# Effect of Natural Zeolite Modification Route on the Catalytic Pyrolysis of Post-Consumer Polystyrene Toward Styrene-Rich Liquid Products

**DOI:** 10.3390/polym18151922

**Published:** 2026-08-05

**Authors:** Joaquin Hernandez-Fernandez, Rafael Gonzalez-Cuello, Rodrigo Ortega-Toro

**Affiliations:** 1 Chemistry Program, Department of Natural and Exact Sciences, University of Cartagena, San Pablo Campus, Cartagena de Indias 130015, Colombia; 2 Department of Natural and Exact Science, Universidad de la Costa, Barranquilla 080002, Colombia; 3 Food Packaging and Shelf-Life Research Group (FP&SL), Food Engineering Program, University of Cartagena, Cartagena de Indias 130015, Colombiarortegap1@unicartagena.edu.co (R.O.-T.)

**Keywords:** catalytic pyrolysis polystyrene, natural zeolites, styrene, plastic waste valorization, circular economy, ANOVA, correlation analysis

## Abstract

The catalytic pyrolysis of post-consumer polystyrene (PS) offers a potential route to obtain styrene-rich liquid fractions from plastic waste. In this study, natural zeolites were modified by thermal activation (AT-ZN), acid treatment (AA-ZN), and protonic ion exchange (H-ZN), and their performance was evaluated under different pyrolysis temperatures (400–500 °C), heating rates (10–20 °C min^−1^), and catalyst loadings (5–10 wt.%). Thermogravimetric analysis indicated that zeolite incorporation shifted the apparent PS degradation profile toward lower temperatures, suggesting that the modified solids altered the polymer’s thermal conversion behavior. Product-yield analysis showed that H-ZN provided the most favorable phase distribution, producing high liquid fractions while limiting solid-residue formation. AT-ZN exhibited an intermediate, comparatively stable response. In contrast, AA-ZN promoted greater solid formation and lower liquid recovery, suggesting that more severe catalytic conditions may favor secondary reactions and the accumulation of carbonaceous residues. Targeted GC–MS analysis revealed that styrene was the dominant aromatic compound among the quantified products, with H-ZN consistently showing the highest styrene proportion in the analyzed liquid fraction. Correlation analysis and ANOVA further indicated that the influence of temperature, catalyst loading, and their interactions depended strongly on the zeolite modification route. Overall, the results demonstrate that the route of modification of the natural zeolite strongly affected its composition, textural properties, acidity distribution, thermal behavior, and catalytic performance during PS pyrolysis. XRF, N_2_ adsorption–desorption, NH_3_-TPD, TGA/DTG, and FTIR characterization showed that AA-ZN exhibited the highest Si/Al ratio and BET surface area, whereas H-ZN presented the highest total acidity and the largest contribution of medium- and strong-acid sites. The combined characterization and pyrolysis results indicate that the preservation of styrene-rich liquid products was governed by the balance between acid-site distribution and pore accessibility, rather than by surface area or total acidity considered in isolation.

## 1. Introduction

The exponential accumulation of plastic waste, particularly polystyrene, has become one of the most pressing environmental challenges of the 21st century. Polystyrene is a widely used aromatic polymer in packaging, insulation materials, and disposable products because of its low cost, light weight, and favorable mechanical performance. However, its high resistance to biological degradation and long environmental persistence make post-consumer PS waste a critical source of plastic pollution [1,2]. Although mechanical recycling remains the most established route for PS waste management, it is frequently associated with downcycling because repeated processing promotes polymer degradation and deterioration of material properties. In this context, chemical recycling via catalytic depolymerization, which converts PS waste into styrene-rich products, has emerged as a strategic route to advance circularity in polystyrene recycling [3,4].

Thermal pyrolysis of PS generally requires high reaction temperatures to promote C–C bond scission and volatilization of styrenic fragments. However, the process often produces complex liquid mixtures whose monomer selectivity is strongly governed by operating conditions such as temperature, residence time, heating rate, and reactor configuration [5,6,7]. To overcome these limitations, catalytic pyrolysis has been proposed as an effective strategy to reduce the thermal severity of PS degradation while improving the quality and selectivity of the resulting liquid products [2,8]. Synthetic zeolites such as HZSM-5 and USY have shown high efficiency in directing polymer-derived vapors toward aromatic hydrocarbons; nevertheless, their broader practical implementation can be limited by high synthesis costs, catalyst deactivation, and the need for regeneration after coke deposition [9,10]. Natural zeolites, owing to their abundance, low cost, and thermal stability, represent sustainable alternatives to synthetic zeolitic catalysts. However, their direct catalytic application is often hindered by compositional heterogeneity, insufficient acidity, and non-optimized pore architecture, which can limit their competitiveness relative to engineered synthetic frameworks [11,12,13].

Despite the growing interest in zeolite-assisted PS pyrolysis, a relevant gap remains in understanding how different modification routes applied to low-cost natural zeolites affect the balance among liquid recovery, solid residue formation, and the preservation of styrenic products. Most studies involving natural zeolites, including those reported by Miandad et al. and Pavlovskyi et al., have primarily focused on improving liquid-fuel production or on the formation of broad aromatic hydrocarbons [14,15]. However, monomer-oriented PS pyrolysis requires a more controlled conversion environment, in which primary depolymerization is favored. At the same time, excessive secondary cracking, hydrogen transfer reactions, dealkylation, and coke-forming pathways are minimized [16,17]. Thermally activated natural zeolites may preserve styrene as a major product, but their moderate catalytic activity can limit their ability to substantially modify product selectivity compared with thermal pyrolysis benchmarks [18].

In this context, the present work compares three modified natural zeolites obtained by thermal activation, acid treatment, and ammonium-ion exchange, followed by calcination to generate the protonic form. The parent and modified materials were characterized by X-ray fluorescence spectroscopy (XRF), N_2_ adsorption–desorption, ammonia temperature-programmed desorption (NH_3_-TPD), thermogravimetric and derivative thermogravimetric analyses (TGA/DTG), and Fourier-transform infrared spectroscopy (FTIR). These complementary analyses were used to determine how each modification route affected the bulk composition, molar Si/Al ratio, textural properties, total acidity, acid-strength distribution, hydration behavior, thermal stability, and characteristic vibrations of the aluminosilicate framework [19,20,21,22,23,24].

From a sustainability perspective, this work offers two main advantages within its experimental scope. First, it employs low-cost natural zeolitic precursors, thereby reducing energy dependence- and reagent-intensive synthetic catalysts [25]. Second, by promoting styrene-rich liquid fractions at comparatively moderate temperatures of 400–450 °C, the process may reduce the thermal severity required for PS conversion relative to conventional high-temperature cracking routes, which typically operate above 600 °C [20]. Accordingly, shifting the process from a waste-to-fuel approach toward a monomer-oriented strategy provides a technically relevant pathway for polystyrene valorization. It supports the development of more sustainable chemical feedstocks from plastic waste [3,4,26,27]. Although the composition, textural properties, acidity distribution, thermal behavior, and vibrational characteristics of the fresh catalysts were directly evaluated, post-reaction catalyst characterization, complete product speciation, direct gas analysis, and regeneration behavior were outside the scope of the present study. Therefore, the work should be interpreted as a physicochemical and catalytic comparison of natural-zeolite modification routes rather than as a complete assessment of catalyst deactivation, regeneration, and long-term structure–activity relationships.

## 2. Methodology

### 2.1. Materials and Feedstock Preparation

The feedstock utilized in this study consisted of post-consumer polystyrene collected from commercial packaging waste, promoting a circular economy approach [1]. The plastic was cleaned, dried, and shredded into particles approximately 2–5 mm in size to ensure uniform heat transfer during pyrolysis. The primary catalyst precursor was a natural zeolite of the clinoptilolite type, selected for its high thermal stability and abundance [11,28].

### 2.2. Catalyst Modification Strategies

The natural zeolite was subjected to three modification protocols to evaluate the effects of thermal activation, acid treatment, and protonic ion exchange on its physicochemical properties and catalytic behavior during post-consumer PS pyrolysis. The resulting materials were denoted as thermally activated zeolite (AT-ZN), acid-treated zeolite (AA-ZN), and protonic-form zeolite (H-ZN). These designations correspond to the applied preparation routes. In contrast, the compositional, textural, acidic, thermal, and vibrational properties of the resulting materials were subsequently evaluated using the characterization procedures described in Section 2.6.

For the thermal activation route, the parent natural zeolite was washed with deionized water, dried at 105 °C, and subsequently calcined in a muffle furnace at 550 °C for 5 h. Thermal treatment is commonly applied to natural zeolites to remove adsorbed water and volatile impurities and to modify the accessibility of the porous network, depending on the mineral composition and treatment conditions [29]. The resulting material was designated AT-ZN.

For the acid-treatment route, the natural zeolite was contacted with a 1.0 M HCl solution at 80 °C for 4 h under constant stirring. Acid treatment of natural zeolites can promote the removal of exchangeable cations and extra-framework species, partial dealumination, and changes in pore accessibility and acid-site distribution, depending on the treatment severity and initial zeolite composition [11]. After treatment, the solid was separated, repeatedly washed with deionized water until the washing solution reached approximately neutral pH, dried at 105 °C, and designated AA-ZN. The compositional, textural, and acidity changes produced by this treatment were subsequently evaluated by XRF, N_2_ adsorption–desorption, NH_3_-TPD, TGA/DTG, and FTIR.

For the protonic-form route, the natural zeolite was subjected to three consecutive ion-exchange cycles using a 1.0 M NH_4_NO_3_ solution at 80 °C. After each exchange cycle, the solid was separated and washed with deionized water. The ammonium-exchanged material was subsequently dried and calcined at 500 °C to promote the decomposition of NH_4_^+^ species and to generate proton-associated acid sites, following commonly reported ion-exchange procedures for zeolite modification [30,31]. The resulting material was designated H-ZN. NH_3_-TPD quantified its total acidity and acid-strength distribution. However, because pyridine-adsorbed FTIR was not performed, Brønsted and Lewis acid sites were not quantified separately.

The three modified materials were used as comparative catalysts to assess the influence of the zeolite modification route on the behavior of PS pyrolysis. Their measured bulk composition, molar Si/Al ratio, surface area, pore properties, total acidity, acid-strength distribution, thermal response, and FTIR characteristics were used to support the interpretation of the catalytic results. Nevertheless, the physicochemical state of the catalysts after pyrolysis was not directly evaluated; therefore, changes associated with coke deposition, pore blockage, acid-site loss, or framework alteration were not quantitatively established.

### 2.3. Experimental Setup and Pyrolysis Procedure

**Figure 1:**
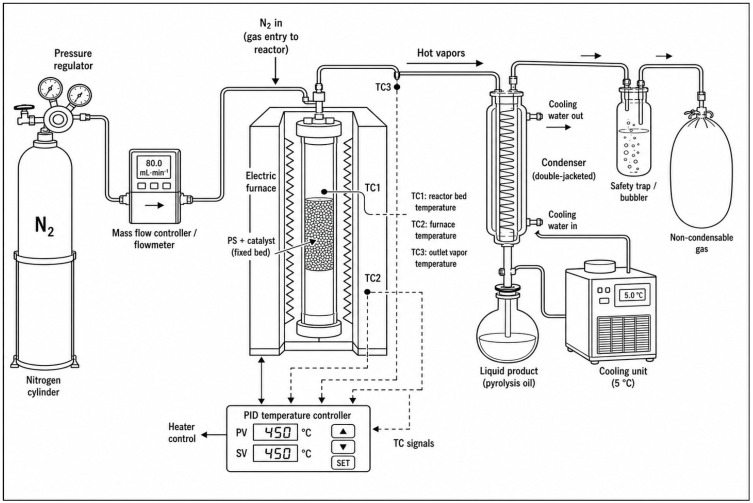
Schematic diagram of the laboratory-scale semi-batch catalytic pyrolysis system used for the conversion of post-consumer polystyrene. The system includes a nitrogen supply cylinder connected to a mass flow controller, a stainless-steel reactor containing a stationary polymer–catalyst charge and placed inside an electrically heated furnace, and a double-jacketed condenser coupled to a cooling unit for liquid-product recovery. The N_2_ inlet is directly connected to the reactor to maintain an inert atmosphere and continuously entrain pyrolysis vapors toward the condensation system. Three thermocouples were incorporated to monitor the reactor-bed temperature (TC1), furnace temperature (TC2), and outlet vapor temperature (TC3). The thermocouple signals were connected to the PID temperature controller for process monitoring and furnace regulation. Condensable vapors were collected as pyrolysis oil, while the non-condensable fraction passed through a safety trap/bubbler before leaving the system.

Catalytic pyrolysis experiments were performed in a laboratory-scale semi-batch reactor with a stationary solid phase and continuous N_2_ sweep. As shown in Figure 1, the setup consisted of a nitrogen carrier-gas line, an electrically heated reactor–furnace assembly, and a downstream condensation system for liquid recovery. High-purity nitrogen, 99.99%, was supplied at 100 mL min^−1^ through a mass flow controller and introduced directly into the reactor inlet to maintain an inert atmosphere and continuously entrain pyrolysis vapors from the reaction zone toward the condensation system. Therefore, although the polymer–catalyst charge remained stationary in the reactor during each experiment, the volatile products were continuously removed by the carrier gas stream. This operating mode is more accurately described as semi-batch rather than as a strict batch configuration.

The reactor was heated using an electric furnace coupled to a PID temperature controller. The system was operated at final temperatures of 400, 450, and 500 °C, with heating rates of 10 and 20 °C min^−1^. Three thermocouples were used to monitor the thermal profile: TC1 measured the reactor-bed temperature, TC2 monitored the furnace temperature, and TC3 monitored the outlet-vapor temperature. These thermocouples were connected to the PID controller to regulate furnace power and ensure stable thermal operation.

For each run, 50 g of post-consumer PS were loaded with the corresponding amount of catalyst. Catalyst loadings of 5 and 10 wt.% relative to PS corresponded to 2.5 and 5.0 g of catalyst, respectively. Before heating, the reactor was purged with N_2_ for 15 min. Once the target temperature was reached, an isothermal residence time of 30 min was maintained. The condensable vapors were recovered in a double-jacketed glass condenser connected to a recirculating bath at 5 °C. At the same time, non-condensable gases passed through a safety trap/bubbler before leaving the system. The remaining solid residue was recovered from the reactor after cooling.

### 2.4. Product-Yield Determination and Pyrolysis Oil Analysis by GC–MS

After each catalytic pyrolysis experiment, the liquid fraction was recovered from the condenser and receiver and weighed gravimetrically. The solid fraction was determined by weighing the material remaining in the reactor after cooling. In catalytic runs, the initial catalyst mass was considered in the solid-residue calculation to avoid overestimating the carbonaceous residue. Therefore, product yields were normalized with respect to the initial PS mass. The liquid yield was calculated from the mass of recovered oil, the solid yield from the corrected reactor residue, and the gaseous fraction by difference according to the following mass–balance relationships:

Liquid yield (wt.%) = (mass of recovered oil/initial mass of PS) × 100

Solid yield (wt.%) = [(final solid residue − initial catalyst mass)/initial mass of PS] × 100

Gas yield (wt.%) = 100 − liquid yield − solid yield

The gas fraction was therefore estimated by difference from the liquid and corrected solid fractions. This approach allowed the product distribution to be expressed in terms of liquid, corrected solid residue, and non-condensable gaseous fractions. Because the gas fraction was calculated by difference, individual gaseous products were not quantified in this study.

The chemical composition of the recovered pyrolysis oil was analyzed by gas chromatography–mass spectrometry (GC–MS), focusing on the identification and relative quantification of the main aromatic compounds generated during PS catalytic pyrolysis. The analysis was performed using an Agilent 7890B GC system equipped with a DB-5MS capillary column (30 m × 0.25 mm × 0.25 µm film thickness; Agilent Technologies, Santa Clara, CA, USA), with helium as the carrier gas. The oven temperature program was initiated at 40 °C and held for 2 min, then ramped to 300 °C at 10 °C min^−1^ [4,32].

The main aromatic compounds, including styrene, toluene, and ethylbenzene, were identified by comparing their mass spectra with the NIST mass spectral library database. Special attention was given to styrene because it represents one of the main target compounds in PS chemical recycling and monomer-oriented depolymerization strategies [4,32]. The relative abundance of styrene, toluene, and ethylbenzene was estimated from their respective chromatographic peak areas and expressed as percentages of the analyzed aromatic fraction of the pyrolysis oil. Therefore, the GC–MS results reported in this study correspond to targeted aromatic composition rather than to a complete molecular mass balance of the liquid product.

### 2.5. Experimental Design and Statistical Analysis

A full factorial experimental design was implemented to evaluate the influence of the main operating variables on the catalytic pyrolysis of post-consumer PS. The operating variables were pyrolysis temperature (three levels: 400, 450, and 500 °C); heating rate (two levels: 10 and 20 °C min^−1^); and catalyst loading (two levels: 5 and 10 wt.%). This 3 × 2 × 2 experimental matrix was applied independently to each zeolite modification route, namely thermally activated zeolite, AT-ZN; protonic zeolite, H-ZN; and acid-activated zeolite, AA-ZN. Therefore, the zeolite modification route was treated as a categorical catalyst group for comparative purposes. In contrast, temperature, heating rate, and catalyst loading were treated as the operating variables in the factorial design. Accordingly, the complete experimental set comprised 36 catalytic pyrolysis conditions, obtained by combining three temperatures, two heating rates, two catalyst loadings, and three catalyst modification routes.

For each condition, product yields were calculated from the gravimetric mass balance, and the aromatic composition of the recovered liquid fraction was determined by targeted GC–MS analysis. Experimental variability was quantified using the sample standard deviation (SD). Summary datasets containing the mean values, SD values, and number of replicates for product yields and GC–MS aromatic composition are provided in Tables S1 and S2, respectively. For most conditions, *n* = 3 was available; the number of replicates used for each condition is indicated in the Supplementary Tables. The data were analyzed using analysis of variance (ANOVA) to determine the significance of the main effects and their interactions on product yields. The high F-values and low *p*-values observed for the H-ZN and AA-ZN catalysts, as discussed in the Results section, confirm that the experimental design effectively captured the sensitivity of the depolymerization process to the evaluated operating factors [33,34,35,36,37].

The product-yield dataset includes the liquid fraction, corrected solid fraction, and gas fraction calculated by difference. Liquid yield was determined from the mass of recovered oil, solid yield was corrected by subtracting the initial catalyst mass from the final reactor residue, and gas yield was calculated as 100 − liquid yield − corrected solid yield. Accordingly, the liquid, solid, and gas values represent the global phase distribution, normalized to the initial PS mass.

The GC–MS dataset includes the relative abundances of styrene, toluene, and ethylbenzene in the aromatic fraction of the pyrolysis oil. These values describe the targeted aromatic composition of the liquid fraction and should not be interpreted as a complete molecular mass balance of the pyrolysis oil. The same summary datasets were used to construct the main figures and to support the discussion of product-yield trends and aromatic-product distribution.

Pearson correlation analysis was also used as an exploratory tool to evaluate associations among operating variables, phase yields, and targeted aromatic composition. Because some response variables correspond to constrained compositional data, particularly the liquid–solid–gas distribution and the relative GC–MS composition, the correlation results were interpreted as descriptive associations rather than as standalone mechanistic proof [38,39,40].

### 2.6. Physicochemical Characterization Methods for the Parent and Modified Natural Zeolites

The parent natural zeolite, ZN, and the modified materials obtained by thermal activation, AT-ZN; acid treatment, AA-ZN; and ammonium ion exchange followed by calcination, H-ZN, were characterized by X-ray fluorescence spectroscopy, XRF; N_2_ adsorption–desorption analysis; ammonia temperature-programmed desorption, NH_3_-TPD; thermogravimetric and derivative thermogravimetric analyses, TGA/DTG; and Fourier-transform infrared spectroscopy, FTIR. These complementary techniques were used to evaluate changes in bulk oxide composition, molar Si/Al ratio, textural properties, acid-site distribution, hydration behavior, thermal stability, and aluminosilicate-framework vibrations.

Bulk oxide composition was determined using a Rigaku ZSX Primus X-ray fluorescence spectrometer (Rigaku Corporation, Akishima, Tokyo, Japan). Powdered samples were homogenized and prepared as fused beads using lithium tetraborate as the flux. The concentrations of SiO_2_, Al_2_O_3_, CaO, Na_2_O, K_2_O, MgO, Fe_2_O_3_, and minor oxides were expressed as weight percentages. Loss on ignition was determined gravimetrically by heating an aliquot of each sample to 1000 °C at 10 °C min^−1^, maintaining this temperature for 1 h, and subsequently cooling the material to room temperature before reweighing. These conditions are directly comparable to procedures reported for natural and HCl-treated clinoptilolite.

The molar Si/Al ratio was calculated from the SiO_2_ and Al_2_O_3_ contents according to:

$$\frac{Si}{Al}=\frac{w_{SiO_{2}}/M_{SiO_{2}}}{2w_{Al_{2}O_{3}}/M_{Al_{2}O_{3}}}$$

where $w_{SiO_{2}}$ and $w_{Al_{2}O_{3}}$ are the mass fractions of SiO_2_ and Al_2_O_3_, respectively, and $M_{SiO_{2}}$ and $M_{Al_{2}O_{3}}$ are their corresponding molar masses.

Textural properties were determined from N_2_ adsorption–desorption isotherms recorded at 77 K using a Micromeritics 3FLEX surface-area and porosity analyzer (Micromeritics Instrument Corporation, Norcross, GA, USA). Before analysis, approximately 0.20 g of each sample was degassed under vacuum at 300 °C for 10 h to remove adsorbed water and weakly retained volatile species. The specific surface area, $S_{BET}$, was calculated using the multipoint Brunauer–Emmett–Teller method within the relative-pressure range $0.05<P/P_{0}<0.35$. The total pore volume was estimated from the nitrogen uptake at $P/P_{0}\approx 0.99$, whereas the micropore area and micropore volume were determined using the t-plot method.

The mean apparent pore diameter was calculated according to:

$$D_{p}=\frac{4V_{total}}{S_{BET}}$$

where $V_{total}$ is the total pore volume and $S_{BET}$ is the BET surface area. The appropriate unit-conversion factor was applied to express $D_{p}$ in nanometers.

The total acidity and acid-strength distribution were evaluated by NH_3_-TPD using a TPDRO 1100 series chemisorption analyzer (Thermo Fisher Scientific S.p.A., Milan, Italy) equipped with a thermal conductivity detector. Approximately 100 mg of each zeolite was initially activated under helium at 400 °C for 1 h using a heating rate of 5 °C min^−1^. The sample was subsequently cooled to 100 °C and exposed to an NH_3_-containing stream until saturation. Physically and weakly adsorbed ammonia was removed by purging with helium until a stable detector baseline was obtained. Desorption was then performed by heating the sample to 700 °C under helium, and the released ammonia was continuously monitored. The use of a TPDRO 1100 instrument, with ammonia adsorption near 100 °C and desorption up to 700 °C, has been reported for ammonium-exchanged and protonated clinoptilolite.

For comparative integration, the NH_3_-TPD profiles were operationally divided into weak-, medium-, and strong-acid-site regions corresponding to 100–250, 250–400, and 400–700 °C, respectively. Total acidity was calculated from the integrated NH_3_ desorption signal and expressed as mmol NH_3_ g^−1^. These intervals represent an operational classification because the boundaries between acid-strength populations may overlap. NH_3_-TPD quantifies the total population and relative strength of acid sites but does not independently distinguish between Brønsted and Lewis acidity.

Thermogravimetric analyses were conducted using a Setaram Setsys Evolution simultaneous thermal analyzer (SETARAM Instrumentation, Caluire, France). Approximately 35 mg of each zeolite was placed in an alumina crucible and heated from 30 to 800 °C at 10 °C min^−1^ under a continuous N_2_ atmosphere. An empty alumina crucible was used as the reference. The residual mass was recorded as a function of temperature. A closely related clinoptilolite study used the same instrument, an N_2_ atmosphere, a sample mass of approximately 35 mg, and a heating rate of 10 °C min^−1^, although its complete scan extended to 1000 °C.

The negative derivative thermogravimetric curve was calculated according to:

$$-DTG=-\frac{dW}{dT}$$

where W is the residual mass percentage and T is the temperature. Mass losses were quantified within the intervals 30–150, 150–350, and 350–800 °C. The first interval was associated mainly with physically adsorbed and channel-confined water; the second, with more strongly retained water and volatile species; and the third, with progressive dehydroxylation and removal of strongly bound species. Studies of natural and acid-treated clinoptilolite report continuous mass loss, dominated by dehydration at lower temperatures, followed by smaller, high-temperature losses attributable to strongly associated water and dehydroxylation.

FTIR spectra were recorded using a Bruker Vertex 80v spectrometer equipped with an RT-DLaTGS detector (Bruker Optics GmbH & Co. KG, Ettlingen, Germany). The conventional KBr-pellet method was used. Before pellet preparation, the zeolite samples and spectroscopic-grade KBr were dried at 100 °C for 3 h. Approximately 0.5 mg of zeolite was mixed with 200 mg of KBr in an agate mortar, and the homogeneous mixture was compressed under a load of approximately 2 tons for 2 min. Spectra were collected between 4000 and 400 cm^−1^ at a resolution of 4 cm^−1^ using 16 accumulated scans at room temperature. These instrument specifications and preparation conditions have been reported for natural and HCl-modified clinoptilolite.

The FTIR interpretation focused on the O–H stretching region between 3650 and 3200 cm^−1^, the H–O–H bending vibration near 1650–1620 cm^−1^, the asymmetric Si–O–Si and Si–O–Al stretching region between 1100 and 1000 cm^−1^, the symmetric framework vibrations at 805–780 cm^−1^, HEU-type ring vibrations at 620–590 cm^−1^, and T–O bending modes between 500 and 450 cm^−1^, where T represents Si or Al. Comparable clinoptilolite spectra contain the principal framework vibration near 1050 cm^−1^, water-related bands near 1638 and 3434 cm^−1^, and bridging hydroxyl contributions near 3630 cm^−1^.

### 2.7. Sustainability and Environmental Considerations

In alignment with green chemistry principles, this methodology emphasizes three main aspects. First, the use of low-cost catalysts avoids the need for expensive synthetic templates by employing naturally occurring minerals [5,34]. Second, optimizing the process at 400–450 °C, a temperature range significantly lower than that used in conventional industrial thermal cracking (typically >600 °C), improves energy efficiency [20]. Third, the recovery of up to 65% styrene monomer contributes to resource recovery by enabling the potential reintegration of waste-derived products into the polymer production chain [3,4].

## 3. Results

### 3.1. Physicochemical Properties of the Parent and Modified Natural Zeolites

The physicochemical characterization confirmed that the thermal, acid, and ion-exchange treatments produced distinguishable changes in the composition, acidity, textural properties, and hydration behavior of the natural zeolite. The parent material, ZN, contained 66.0 wt.% SiO_2_ and 12.5 wt.% Al_2_O_3_, corresponding to a molar Si/Al ratio of 4.49. Thermal activation produced only a marginal change in this ratio, from 4.49 to 4.51, indicating that calcination did not substantially alter the bulk aluminosilicate composition. The most evident change in AT-ZN was a reduction in loss on ignition from 10.0 to 6.0 wt.%, consistent with the removal of adsorbed water and volatile species during thermal treatment.

As shown in Table 1, acid treatment generated the largest compositional modification. The SiO_2_ content increased to 78.5 wt.%, whereas the Al_2_O_3_ content decreased to 9.5 wt.%, increasing the molar Si/Al ratio to 7.02. Simultaneously, the CaO, Na_2_O, K_2_O, and MgO contents decreased markedly relative to those of ZN. These results provide experimental evidence of partial dealumination and decationization during HCl treatment. In contrast, H-ZN exhibited an intermediate Si/Al ratio of 5.07, together with lower Na_2_O, K_2_O, CaO, and MgO contents than the parent material (Table 1). This composition is consistent with the removal of exchangeable cations during the NH_4_^+^-exchange procedure and the subsequent formation of the protonic material after calcination.

The modification routes also produced different acidity distributions. ZN exhibited a total acidity of 0.35 mmol NH_3_ g^−1^, distributed among weak, medium, and strong sites at 0.18, 0.12, and 0.05 mmol g^−1^, respectively. Thermal activation decreased the total acidity to 0.28 mmol NH_3_ g^−1^, mainly because of the reduction in weak acid sites. AA-ZN showed a higher total acidity of 0.55 mmol NH_3_ g^−1^, with increased medium- and strong-acid contributions of 0.23 and 0.15 mmol g^−1^. The highest total acidity was measured for H-ZN, 0.72 mmol NH_3_ g^−1^, including 0.31 mmol g^−1^ of medium sites and 0.25 mmol g^−1^ of strong sites. Thus, proton exchange increased not only the total amount of adsorbed ammonia but particularly the population of medium- and high-strength acid sites.

These measurements provide direct support for differentiating catalytic materials by their acid-site distributions. Nevertheless, the NH_3_-TPD results should not be used to assign Brønsted and Lewis acid-site concentrations independently, since this distinction would require complementary characterization using pyridine-adsorbed FTIR or another site-selective probe (Thermo Fisher Scientific S.p.A., Milan, Italy).

As shown in Table 2, the textural analysis further demonstrated that the treatments affected pore accessibility. ZN exhibited a BET surface area of 24 m^2^ g^−1^, which increased to 32 m^2^ g^−1^ after thermal activation, 68 m^2^ g^−1^ after acid treatment, and 46 m^2^ g^−1^ after proton exchange. Consequently, AA-ZN exhibited the highest total pore volume (0.145 cm^3^ g^−1^) and micropore area (25 m^2^ g^−1^), whereas H-ZN showed intermediate values of 0.105 cm^3^ g^−1^ and 20 m^2^ g^−1^, respectively. The mean apparent pore diameter decreased from 12.5 nm for ZN to 10.6, 8.5, and 9.1 nm for AT-ZN, AA-ZN, and H-ZN, respectively (Table 2).

The modification treatments also altered the textural properties of the natural zeolite. As summarized in Table 3, the parent zeolite exhibited a BET surface area of 24 m^2^ g^−1^, which increased to 32 m^2^ g^−1^ after thermal activation, 46 m^2^ g^−1^ after proton exchange, and 68 m^2^ g^−1^ after acid treatment. The increase was accompanied by changes in micropore area, total pore volume, micropore volume, and mean pore diameter, confirming that each treatment modified pore accessibility to varying degrees.

AA-ZN showed the greatest textural development, with a BET surface area of 68 m^2^ g^−1^ and a total pore volume of 0.145 cm^3^ g^−1^, representing increases of approximately 183% and 93%, respectively, relative to ZN. These changes are consistent with the removal of exchangeable cations, partial dealumination, and elimination of pore-blocking species during acid treatment. H-ZN exhibited intermediate textural properties, with a BET surface area of 46 m^2^ g^−1^ and a micropore area of 20 m^2^ g^−1^, whereas AT-ZN showed a more moderate increase in surface area and pore volume. The decrease in mean pore diameter after modification should not be interpreted as pore collapse because it occurred simultaneously with increases in surface area and pore volume. Instead, it likely reflects a greater contribution from smaller accessible pores to the calculated pore-size distribution.

The textural results should be interpreted together with the NH_3_-TPD measurements. AA-ZN exhibited the highest surface area, whereas H-ZN showed the highest total acidity and the largest contribution from medium and strong acid sites. Therefore, the catalytic behavior cannot be attributed exclusively to either surface area or acidity; rather, it depends on the combined effects of pore accessibility and acid-site distribution.

The combined acidity and BET results indicate that catalytic behavior cannot be explained by surface area alone. AA-ZN possessed the highest BET area and pore volume, whereas H-ZN exhibited the highest total acidity and the largest contribution of medium and strong acid sites. This distinction is relevant to the pyrolysis results: the high liquid recovery and styrene proportion obtained with H-ZN can be attributed to a balance between accessible porosity and acid functionality, whereas the higher solid formation observed with AA-ZN occurred despite its larger surface area. Therefore, surface development alone did not guarantee improved liquid-product selectivity.

Thermogravimetric analysis of the fresh materials showed that the largest mass loss occurred between 30 and 150 °C and was primarily associated with the removal of physically adsorbed and weakly retained water. ZN exhibited an 8.5% loss in this interval, compared with 3.2% for AT-ZN, 3.8% for AA-ZN, and 5.0% for H-ZN. The lower low-temperature losses of the modified materials confirm that the treatments altered their hydration state. Additional losses between 150 and 350 °C were attributed to more strongly retained water and residual volatile species, whereas the smaller losses between 350 and 800 °C were consistent with progressive dehydroxylation.

**Figure 2:**
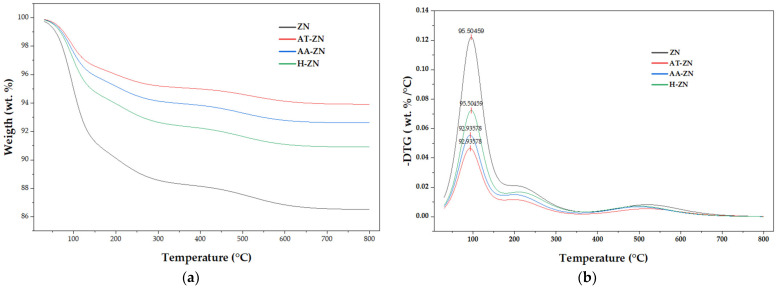
Thermal characterization of the parent and modified natural zeolites. (**a**) TGA profiles of ZN, AT-ZN, AA-ZN, and H-ZN between 30 and 800 °C. (**b**) Corresponding DTG profiles showing the temperature regions associated with water desorption, removal of more strongly retained species, and progressive dehydroxylation.

As shown in Figure 2 and summarized in Table 4, the total mass losses followed the order ZN (13.5%) > H-ZN (9.1%) > AA-ZN (7.4%) > AT-ZN (6.1%). The comparatively low mass loss of AT-ZN reflects the prior calcination treatment. In contrast, the intermediate mass loss observed for H-ZN is consistent with its greater population of hydroxyl-associated and protonic sites. No major high-temperature decomposition event was detected in the TGA or DTG profiles, confirming that all materials retained substantial thermal stability over the temperature range relevant to PS pyrolysis.

The FTIR spectra retained the principal bands associated with the clinoptilolite-type aluminosilicate framework. The broad band between 3500 and 3200 cm^−1^ and the deformation band between 1650 and 1620 cm^−1^ were assigned to adsorbed water. Their lower relative contributions in AT-ZN and AA-ZN were consistent with the reduced low-temperature mass loss observed by TGA. The band between 3650 and 3600 cm^−1^, associated with structural hydroxyl groups or bridging Si–OH–Al environments, was more perceptible for H-ZN after proton exchange.

**Figure 3:**
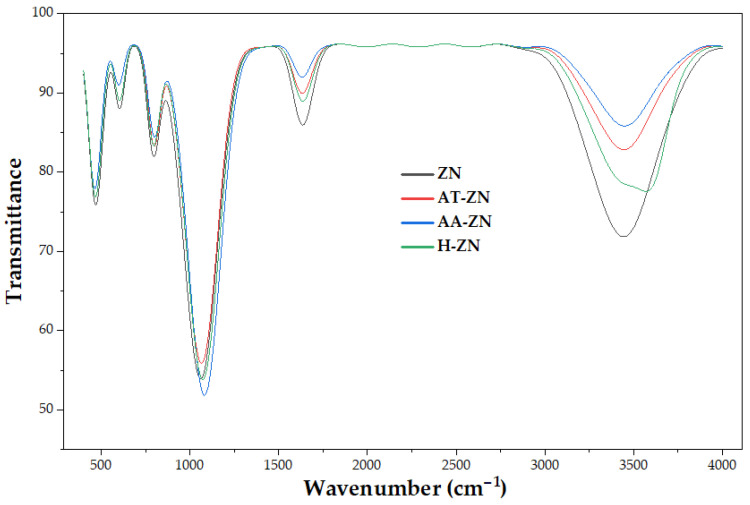
FTIR spectra of the parent natural zeolite, ZN, and the thermally activated, AT-ZN, acid-treated, AA-ZN, and proton-exchanged, H-ZN, materials. Spectra are vertically offset for clarity.

As shown in Figure 3, the intense framework band in the 1100–1000 cm^−1^ region, assigned to the asymmetric stretching vibrations of Si–O–Si and Si–O–Al bonds, remained evident in all samples, indicating that the principal aluminosilicate framework was preserved after modification. AA-ZN exhibited the most pronounced relative alteration in this spectral region, consistent with its higher Si/Al ratio and the occurrence of partial dealumination. The bands at 805–780, 620–590, and 500–450 cm^−1^, assigned to symmetric framework stretching, HEU-ring vibrations, and T–O bending modes, respectively, were also retained, although moderate changes were observed following acid treatment.

The FTIR spectra of the parent and modified natural zeolites retained the principal absorption bands associated with the clinoptilolite-type aluminosilicate framework. The assignments of the main spectral regions and the qualitative changes produced by thermal activation, acid treatment, and proton exchange are summarized in Table 5.

The broad absorption region between 3500 and 3200 cm^−1^, together with the band at 1650–1620 cm^−1^, was associated with O–H stretching and H–O–H bending vibrations of adsorbed and channel-confined water, respectively. Both contributions decreased after thermal activation, consistent with the lower mass loss observed for AT-ZN below 150 °C. AA-ZN also exhibited a lower contribution from water-related bands, which agrees with the removal of exchangeable cations and the resulting modification of the hydration environment during acid treatment.

The weak contribution between 3650 and 3600 cm^−1^ was assigned to structural hydroxyl groups and bridging Si–OH–Al environments. This region was more perceptible for H-ZN, consistent with the generation of proton-associated hydroxyl environments after NH_4_^+^ exchange and subsequent calcination. Nevertheless, this assignment should be considered qualitative because conventional FTIR does not independently quantify Brønsted acidity.

The intense band within 1100–1000 cm^−1^ corresponded to asymmetric stretching vibrations of Si–O–Si and Si–O–Al linkages. Its preservation in all samples indicates that the principal aluminosilicate framework remained present after modification. The displacement toward higher wavenumbers observed for AA-ZN is consistent with the increase in the Si/Al ratio determined by XRF, because partial dealumination increases the relative contribution of Si–O–Si environments. The lower intensity of the bands at 805–780 and 620–590 cm^−1^ for AA-ZN also indicates that acid treatment produced more pronounced local changes in the zeolitic framework than thermal activation or proton exchange.

Finally, the band between 500 and 450 cm^−1^, assigned to T–O bending vibrations, remained intense for ZN, AT-ZN, and H-ZN but was moderately modified for AA-ZN. Together with the preservation of the principal framework bands, these results indicate that the modification treatments altered hydration, hydroxyl environments, and local Si–O–Al connectivity without eliminating the characteristic aluminosilicate structure.

### 3.2. Thermogravimetric Evaluation of PS Degradation over Modified Natural Zeolites

**Figure 4:**
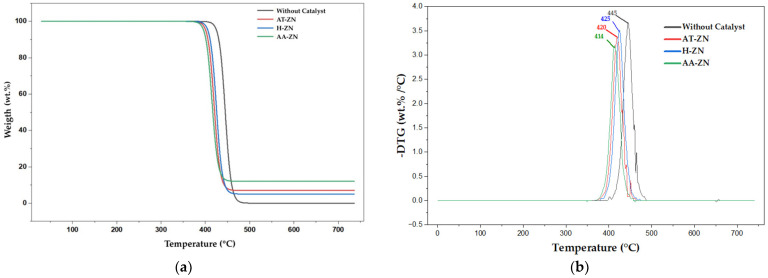
Thermogravimetric evaluation of neat polystyrene and polystyrene mixed with modified natural zeolites. (**a**) TGA profiles showing the residual mass as a function of temperature for PS without catalyst and PS containing thermally activated zeolite, AT-ZN; proton-exchanged zeolite, H-ZN; and acid-treated zeolite, AA-ZN. (**b**) Corresponding negative derivative thermogravimetric curves, −DTG, showing the temperature of maximum mass-loss rate, $T_{max}$.

The thermogravimetric and derivative thermogravimetric profiles presented in Figure 4 show that neat PS and the PS–zeolite mixtures underwent a predominantly single-stage mass-loss process. Neat PS remained thermally stable over most of the initial temperature range, followed by rapid decomposition between approximately 400 and 480 °C. The corresponding −DTG curve exhibited a single maximum at 445 °C, confirming that PS degradation was dominated by one principal volatilization event. This behavior is consistent with the radical-mediated decomposition of the carbon–carbon backbone, which generates styrene, styrenic oligomers, and lower-molecular-weight aromatic fragments [6,7,21,29].

The addition of the modified natural zeolites displaced both the TGA mass-loss region and the −DTG maximum toward lower temperatures. The maximum degradation rate occurred at 425 °C for H-ZN, 420 °C for AT-ZN, and 414 °C for AA-ZN. Relative to neat PS, these values correspond to decreases of approximately 20, 25, and 31 °C, respectively. Therefore, AA-ZN produced the largest shift in the principal degradation event, followed by AT-ZN and H-ZN. The absence of clearly separated secondary DTG maxima indicates that zeolite incorporation did not generate additional independent degradation stages but instead modified the temperature and rate of the dominant PS decomposition process.

The lower $T_{max}$ values observed for the catalytic mixtures demonstrate that the modification route affected the apparent thermal conversion behavior of PS. Zeolite-assisted PS degradation has been associated with interactions between polymer-derived fragments and acidic sites, followed by β-scission, hydrogen transfer, and rearrangement reactions [16,22,35]. The differences among AA-ZN, AT-ZN, and H-ZN demonstrate that the apparent thermal response of PS was governed by the combined effects of acidity and pore accessibility rather than by the presence of the zeolite phase alone [16,19,24]. AA-ZN, which exhibited the highest BET surface area, 68 m^2^ g^−1^, the largest total pore volume, 0.145 cm^3^ g^−1^, and a molar Si/Al ratio of 7.02, produced the greatest displacement of the PS degradation event toward lower temperature. Its total acidity of 0.55 mmol NH_3_ g^−1^, including 0.23 and 0.15 mmol g^−1^ of medium- and strong-acid sites, respectively, further indicates that acid treatment generated a comparatively accessible and catalytically active surface. This behavior is consistent with previous reports showing that acid treatment can increase polymer-cracking activity by removing exchangeable cations, promoting partial dealumination, and modifying the accessibility of acid sites [16,30].

H-ZN exhibited the highest total acidity, 0.72 mmol NH_3_ g^−1^, and the largest combined contribution of medium- and strong-acid sites, whereas its BET surface area, 46 m^2^ g^−1^, remained lower than that of AA-ZN. AT-ZN showed the lowest total acidity (0.28 mmol NH_3_ g^−1^) and a more limited surface-area increase to 32 m^2^ g^−1^. These results explain why the thermal displacement did not follow a simple order based exclusively on total acidity: AA-ZN combined greater accessible surface development with substantial medium and strong acidity, while H-ZN provided a more acidic but less extensively developed porous structure. Nevertheless, a larger shift toward a lower degradation temperature does not necessarily imply higher selectivity for styrene-rich liquids, as a more severe catalytic environment can also intensify secondary cracking and residue-forming reactions [19,22].

AA-ZN exhibited the lowest $T_{max}$, indicating the strongest effect on the apparent degradation temperature of PS. However, a greater displacement toward lower temperature does not necessarily imply improved recovery of styrene-rich liquids. More extensive catalytic activation may also intensify secondary cracking, hydrogen-transfer reactions, condensation, or the formation of coke precursors [19,22]. Consequently, the TGA and DTG results identify changes in the thermal degradation kinetics but cannot independently establish the chemical composition or selectivity of the resulting products.

Above approximately 500 °C, neat PS approached complete volatilization, whereas the catalytic mixtures retained stable residual fractions. These final residues were approximately 5–7 wt.% for the AT-ZN and H-ZN systems and about 12 wt.% for AA-ZN. Most of this residual mass can be assigned to the thermally stable inorganic zeolite fraction included in each PS–catalyst mixture. A possible contribution from carbonaceous deposits cannot be excluded, particularly for AA-ZN, but it cannot be quantified from the present TGA profiles without analyzing the recovered catalyst separately after pyrolysis.

### 3.3. Effect of Zeolite Type, Zeolite Quantity, Temperature, and Heating Rate on Thermal Pyrolysis Yields

**Figure 5:**
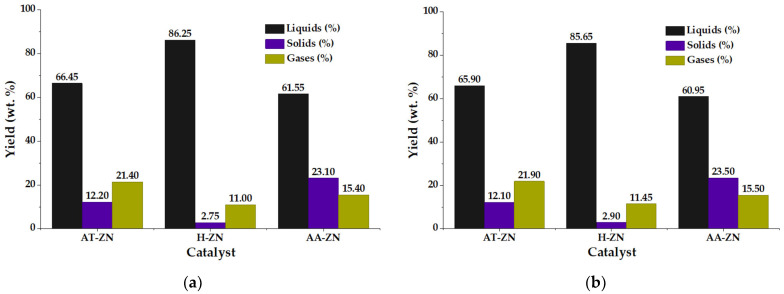

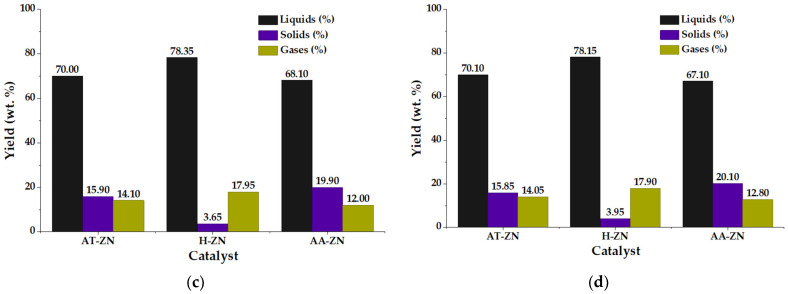
Product-yield distribution from catalytic pyrolysis of polystyrene at 400 °C using AT-ZN, H-ZN, and AA-ZN under equivalent catalyst-loading and heating-rate conditions: (**a**) 5 wt.% catalyst and 10 °C min^−1^; (**b**) 5 wt.% catalyst and 20 °C min^−1^; (**c**) 10 wt.% catalyst and 10 °C min^−1^; and (**d**) 10 wt.% catalyst and 20 °C min^−1^. Liquid, corrected solid, and gas fractions are expressed as wt.% relative to the initial PS mass. The gas fraction was calculated by difference.

**Figure 6:**
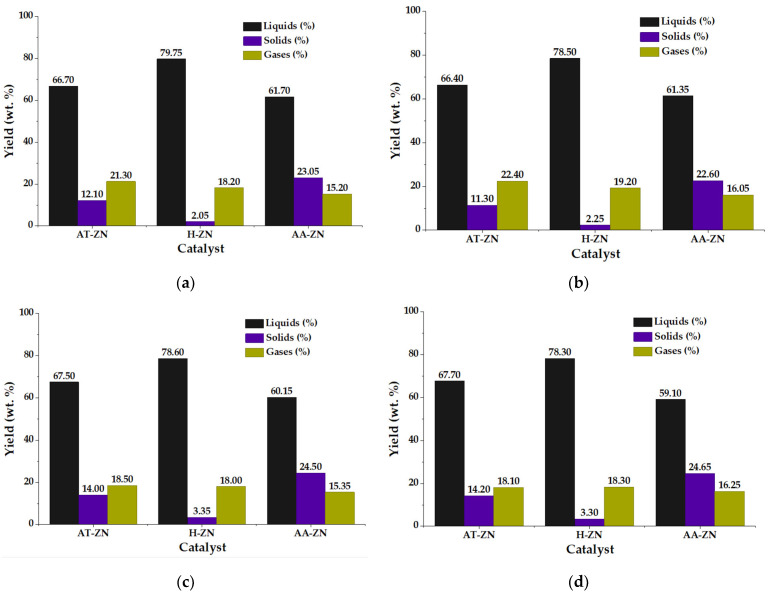
Product-yield distribution from catalytic pyrolysis of polystyrene at 450 °C using AT-ZN, H-ZN, and AA-ZN under equivalent catalyst-loading and heating-rate conditions: (**a**) 5 wt.% catalyst and 10 °C min^−1^; (**b**) 5 wt.% catalyst and 20 °C min^−1^; (**c**) 10 wt.% catalyst and 10 °C min^−1^; and (**d**) 10 wt.% catalyst and 20 °C min^−1^. Liquid, corrected solid, and gas fractions are expressed as wt.% relative to the initial PS mass. The gas fraction was calculated by difference.

Figure 5 and Figure 6 summarize the product-yield distribution obtained during the catalytic pyrolysis of post-consumer PS at 400, 450, and 500 °C, respectively. To avoid repetitive panel-by-panel interpretation, the results are discussed by comparing catalytic behavior across temperature levels under equivalent catalyst loading and heating rate conditions. Overall, the product distribution was governed primarily by the zeolite modification route, whereas the effect of heating rate was comparatively minor within the evaluated range of 10–20 °C min^−1^. This trend agrees with previous studies indicating that catalyst properties and reaction temperature generally exert stronger effects than moderate changes in heating rate during PS pyrolysis [3,16,19,24,33,36].

H-ZN consistently produced the most favorable phase distribution across the three temperatures, combining high liquid recovery with low corrected solid residue. This behavior can be related to its total acidity of 0.72 mmol NH_3_ g^−1^, the highest among the evaluated materials, and its comparatively large contributions from medium- and strong-acid sites, 0.31 and 0.25 mmol g^−1^, respectively. Its BET surface area of 46 m^2^ g^−1^ and total pore volume of 0.105 cm^3^ g^−1^ provided an intermediate level of pore accessibility. The resulting balance between accessible porosity and acid functionality favored the conversion of PS into condensable products without producing the extensive solid accumulation observed for AA-ZN [3,14,16,30]. At higher temperatures or catalyst loading, however, the same acid functionality could promote secondary cracking of condensable vapors, explaining the slight decrease in liquid recovery observed under the most severe conditions [16,19,22,24].

AT-ZN showed an intermediate and comparatively stable response. Thermal activation increased its BET surface area only moderately, from 24 to 32 m^2^ g^−1^, while its total acidity decreased from 0.35 to 0.28 mmol NH_3_ g^−1^. This comparatively low acid-site density and limited textural development are consistent with the lower sensitivity of AT-ZN to changes in temperature, heating rate, and catalyst loading. The material therefore modified PS conversion without strongly intensifying either liquid-selective depolymerization or residue-forming secondary reactions [14,15,29].

AA-ZN exhibited the least favorable profile for liquid recovery, particularly at elevated temperature and catalyst loading. XRF confirmed partial dealumination and decationization, with an increase in the molar Si/Al ratio from 4.49 for ZN to 7.02 for AA-ZN. The acid-treated material also exhibited the highest BET surface area, 68 m^2^ g^−1^, and total pore volume, 0.145 cm^3^ g^−1^, together with a total acidity of 0.55 mmol NH_3_ g^−1^. Although these properties enhanced the apparent thermal activation of PS, the product distribution indicates that the resulting combination of accessibility and acid strength also intensified secondary conversion and solid-residue formation. Therefore, the largest surface area and the greatest reduction in PS degradation temperature did not translate into the highest liquid yield, confirming that catalytic performance depended on the balance between primary depolymerization and secondary cracking or condensation pathways [16,19,22].

**Figure 7:**
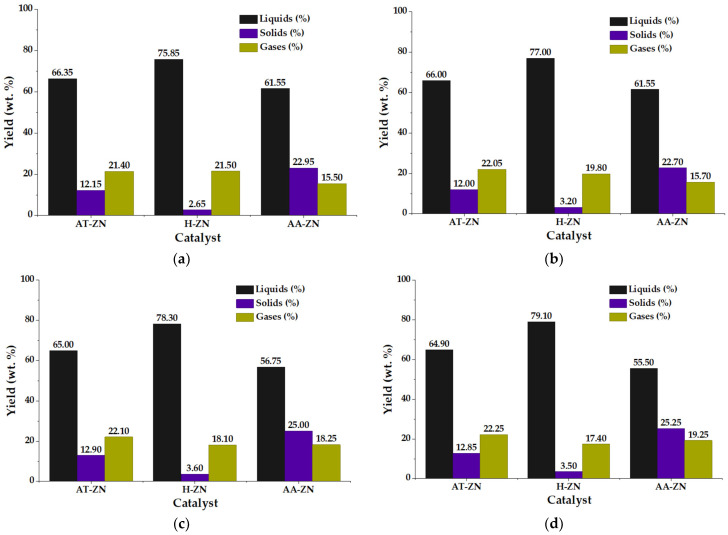
Product-yield distribution from catalytic pyrolysis of polystyrene at 500 °C using AT-ZN, H-ZN, and AA-ZN under equivalent catalyst-loading and heating-rate conditions: (**a**) 5 wt.% catalyst and 10 °C min^−1^; (**b**) 5 wt.% catalyst and 20 °C min^−1^; (**c**) 10 wt.% catalyst and 10 °C min^−1^; and (**d**) 10 wt.% catalyst and 20 °C min^−1^. Liquid, corrected solid, and gas fractions are expressed as wt.% relative to the initial PS mass. The gas fraction was calculated by difference.

Taken together, Figure 5, Figure 6 and Figure 7 show that increasing temperature mainly intensified catalyst-dependent differences rather than producing a simple monotonic improvement in liquid yield. H-ZN was the most effective catalyst for liquid production, AT-ZN showed moderate and stable behavior, and AA-ZN favored higher solid formation. Since the heating-rate effect was small at each fixed temperature, the discussion was streamlined to emphasize cross-temperature comparisons and catalyst-dependent trends. Detailed graphing means, standard deviations, and replicate numbers supporting the product-yield distributions are provided in Table S1 [33,36,39].

The reproducibility and internal consistency of the product-yield data were assessed from the repeated experiments summarized in Table S1. For each operating condition, the liquid fraction was determined gravimetrically, the solid fraction was corrected by subtracting the initial catalyst mass from the final reactor residue, and the gas fraction was calculated by difference from the liquid and corrected solid fractions. Therefore, the liquid, corrected solid, and gas values represent a global phase distribution normalized to the initial PS mass. Because the gas fraction was not directly quantified, the phase balance was closed before finalizing the mean values and graphical labels. This procedure was used to ensure consistency between the gravimetric calculations, the values reported in the main text, and the product-yield distributions shown in Figure 3, Figure 4 and Figure 5. The standard deviations reported in Table S1 indicate that the main catalytic trends were reproducible within the evaluated experimental domain. However, some operating conditions showed higher dispersion, particularly when catalyst loading and temperature jointly intensified product redistribution [33,36,39].

### 3.4. Correlation Analysis of Operating Variables and Product-Yield Distribution

**Figure 8:**
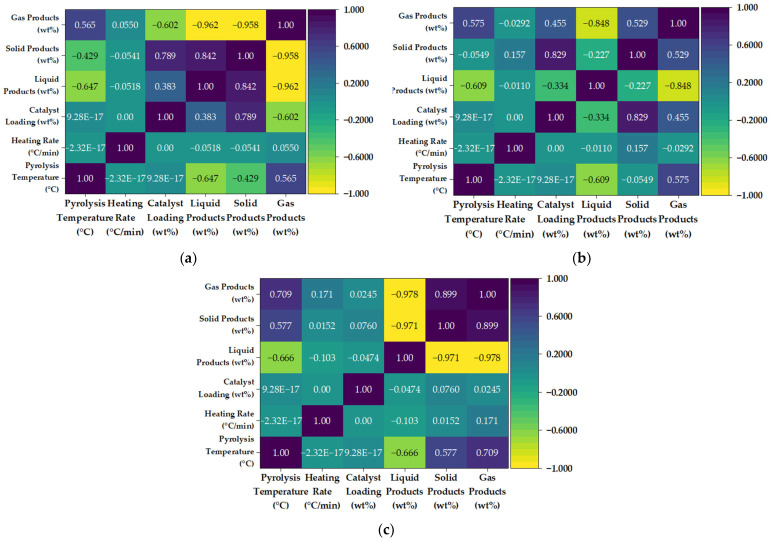
Heatmaps of the Pearson correlation matrices for the catalytic pyrolysis of polystyrene using modified natural zeolites: (**a**) thermally activated zeolite, AT-ZN; (**b**) protonic zeolite, H-ZN; and (**c**) acid-activated zeolite, AA-ZN.

The Pearson correlation matrices in Figure 8 provide a comparative view of how the operating variables influence the distribution of liquid, solid, and gaseous products for each modified zeolite. For AT-ZN (Figure 8a), the strongest relationship was the inverse correlation between liquid and gas products, indicating that gas formation occurs mainly at the expense of the condensable liquid fraction. This behavior is consistent with the competitive nature of PS pyrolysis, in which primary volatile fragments may either condense into liquid-range aromatic products or undergo secondary cracking into lighter, non-condensable species [3,16,19]. Pyrolysis temperature showed a negative correlation with liquid yield and a positive correlation with gas production, suggesting that higher thermal severity promotes secondary cracking of volatile intermediates [5,21,29]. Catalyst loading, in contrast, was positively associated with both liquid and solid fractions and negatively associated with gas production, indicating that additional AT-ZN favors the retention or stabilization of heavier condensable products rather than excessive gas formation [14,15]. The heating rate showed negligible correlations with the product fractions, confirming its limited influence within the evaluated range and suggesting that, for AT-ZN, product distribution is governed more by catalyst surface chemistry than by the thermal ramp [5,33,36,37].

For H-ZN (Figure 8b), the matrix confirms a liquid-selective catalytic behavior but also reveals that this selectivity is sensitive to temperature and catalyst amount. The strong negative correlation between liquid and gas products indicates competition between liquid recovery and secondary cracking, a behavior commonly associated with the transformation of primary styrenic vapors into lighter non-condensable products under more severe catalytic conditions [3,16,19]. Temperature was negatively correlated with liquid yield and positively correlated with gas formation, evidence that increased thermal energy progressively shifts the process toward lighter products [21,22,29]. Catalyst loading was positively correlated with the solid and gas fractions but negatively correlated with the liquid yield, suggesting that excessive H-ZN may intensify secondary reactions or promote the retention of non-volatile residues [16,19,24]. This agrees with the experimental trend observed in the yield figures, where H-ZN was highly efficient for liquid production but did not necessarily benefit from higher catalyst loading.

For AA-ZN (Figure 8c), the correlation pattern was more severe. Liquid yield exhibited very strong negative correlations with both solid and gas products, while solid and gas fractions were strongly and positively correlated with each other. This indicates that, for the acid-activated zeolite, the decrease in liquid recovery is coupled simultaneously with residue formation and gas generation [16,19,32]. Temperature was the main operational variable associated with this behavior, showing positive correlations with both solid and gas fractions and a negative correlation with liquid yield. In contrast, catalyst loading showed little correlation with product distribution, suggesting that the AA-ZN surface may already provide sufficient acid functionality and that increasing the catalyst amount does not improve selectivity [16,22,24].

### 3.5. Analysis of Variance of Product Yields as a Function of Catalyst Modification Route

The ANOVA results for the AT-ZN catalyst are presented in Table 6. The overall model was not statistically significant, as indicated by a model *p*-value of 0.9755, which is well above the conventional significance threshold of α = 0.05. This indicates that, within the evaluated experimental domain, pyrolysis temperature, heating rate, catalyst loading, and their interactions did not explain a statistically meaningful fraction of the variability in product yields. This interpretation is also supported by the distribution of the sum of squares, in which the error contribution was substantially higher than the model contribution, suggesting that most of the variability was attributable to residual dispersion rather than to the controlled factors [38].

Individually, none of the main effects were statistically significant. Temperature showed a *p*-value of 0.5378, indicating that increasing the process temperature within the studied range did not significantly affect the overall product yield distribution for AT-ZN. Similarly, heating rate showed a very weak effect, with a low F-value and a high *p*-value, confirming that the transition from 10 to 20 °C min^−1^ did not substantially alter the process response. Catalyst loading was also non-significant, suggesting that increasing the AT-ZN amount from 5 to 10 wt.% did not yield a statistically significant improvement in product yields. The interaction terms were likewise non-significant, including temperature × heating rate, temperature × catalyst loading, heating rate × catalyst loading, and the three-factor interaction. This indicates that the effect of one factor was independent of the level of another factor within the evaluated range. From a mechanistic perspective, this behavior suggests that AT-ZN operates under a relatively stable catalytic regime, in which moderate changes in temperature, heating rate, or catalyst loading do not substantially alter the balance among liquid, solid, and gaseous products [38,39,40].

In contrast to AT-ZN, the ANOVA results for AA-ZN reveal a statistically significant and highly sensitive catalytic response (Table 7). The overall model was significant, with a *p*-value lower than 0.0001 and a high F-value, indicating that the selected operational variables and their interactions explain most of the variability in product yields. This behavior suggests that acid activation generated a catalytic surface whose activity is strongly modulated by the reaction conditions, particularly temperature and catalyst loading [16,19,22].

Temperature was one of the dominant factors, showing a highly significant effect on product distribution. This confirms that for AA-ZN, increasing thermal severity markedly alters the balance among liquid, solid, and gaseous fractions [29,36,39]. The strongest contribution, however, corresponded to the temperature × catalyst loading interaction, which exhibited the highest sum of squares and an extremely significant *p*-value. This interaction indicates that the effect of catalyst amount depends strongly on the operating temperature. Therefore, increasing the AA-ZN loading does not produce a uniform improvement in liquid yield; instead, under more severe thermal conditions, the higher density of acid sites may intensify secondary cracking, condensation, or coke-forming pathways [16,19,24].

Heating rate and catalyst loading were also statistically significant, although their individual contributions were lower than those of temperature and the temperature × catalyst loading interaction. The significance of heating rate suggests that, for AA-ZN, the residence time and contact between volatile PS fragments and acid sites influence the final mass distribution [33,36,39]. Likewise, the significant heating rate × catalyst loading interaction indicates that the volatilization rate and catalyst availability act jointly to affect the extent of secondary reactions within the catalyst bed [16,22,24].

The ANOVA results for H-ZN are presented in Table 8 and reveal a statistically significant catalytic response. The overall model was highly significant, with a *p*-value lower than 0.0001 and a high F-value, indicating that the selected operational variables explain most of the variability in product yields. Unlike AT-ZN, whose response was only weakly affected by the evaluated factors, H-ZN exhibited more condition-sensitive behavior, consistent with a catalyst whose protonic sites actively participate in the depolymerization and redistribution of PS degradation products [16,30].

Among the evaluated factors, temperature and catalyst loading were both highly significant, confirming that the catalytic performance of H-ZN depends strongly on the thermal environment and the amount of available active sites [22,24,36,37]. However, the most influential term was the temperature × catalyst loading interaction, which exhibited the highest F-value and a *p*-value below 0.0001. This indicates that the effect of catalyst loading is not independent of temperature. In practical terms, increasing the amount of H-ZN may favor liquid production under suitable thermal conditions. However, at higher temperatures, it can also intensify secondary cracking of condensable vapors, shifting part of the product distribution toward lighter fractions [16,19,24].

The heating rate also showed a significant effect, although its contribution was lower than that of temperature and catalyst loading. In addition, the significant heating rate × catalyst loading interaction suggests that the rate of volatile generation and the availability of catalytic sites jointly influence the final product distribution [33,36,39]. By contrast, the temperature × heating rate interaction and the three-factor interaction were not significant, indicating that the most relevant combined effect is specifically associated with the coupling between thermal severity and catalyst amount.

### 3.6. GC–MS Analysis of Aromatic Hydrocarbons in the Pyrolysis Oil

**Figure 9:**
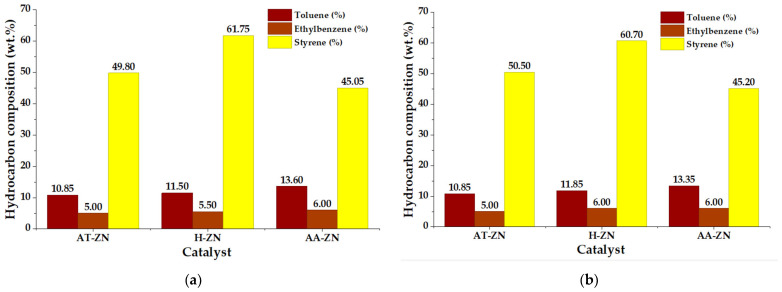

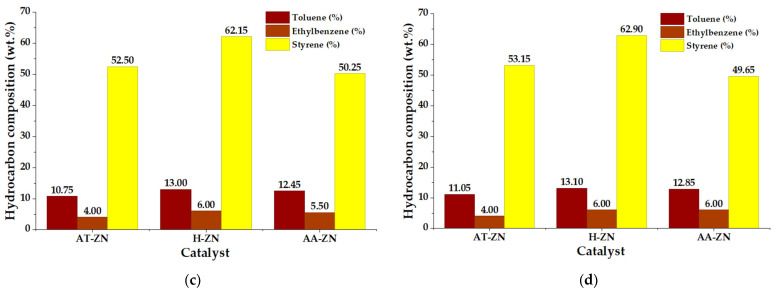
Targeted GC–MS aromatic composition of the pyrolysis oil obtained at 400 °C using AT-ZN, H-ZN, and AA-ZN under equivalent catalyst-loading and heating-rate conditions: (**a**) 5 wt.% catalyst and 10 °C min^−1^; (**b**) 5 wt.% catalyst and 20 °C min^−1^; (**c**) 10 wt.% catalyst and 10 °C min^−1^; and (**d**) 10 wt.% catalyst and 20 °C min^−1^. Toluene, ethylbenzene, and styrene are expressed as relative abundance values within the analyzed aromatic fraction.

**Figure 10:**
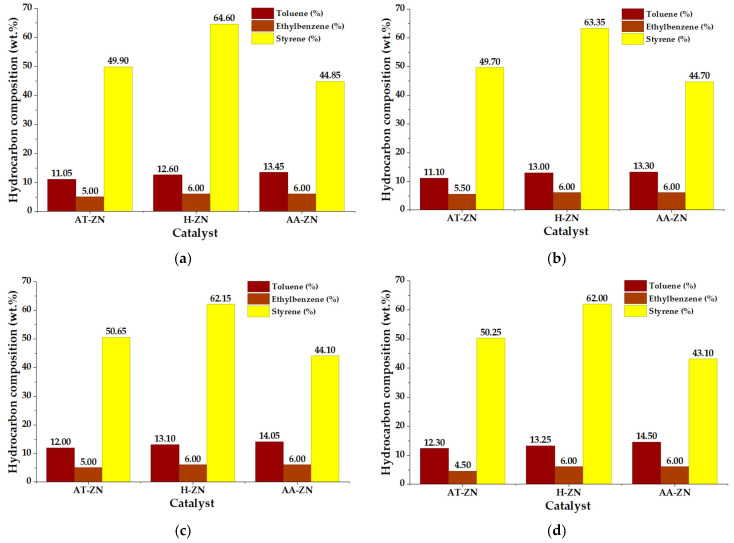
Targeted GC–MS aromatic composition of the pyrolysis oil obtained at 450 °C using AT-ZN, H-ZN, and AA-ZN under equivalent catalyst-loading and heating-rate conditions: (**a**) 5 wt.% catalyst and 10 °C min^−1^; (**b**) 5 wt.% catalyst and 20 °C min^−1^; (**c**) 10 wt.% catalyst and 10 °C min^−1^; and (**d**) 10 wt.% catalyst and 20 °C min^−1^. Toluene, ethylbenzene, and styrene are expressed as relative abundance values within the analyzed aromatic fraction.

**Figure 11:**
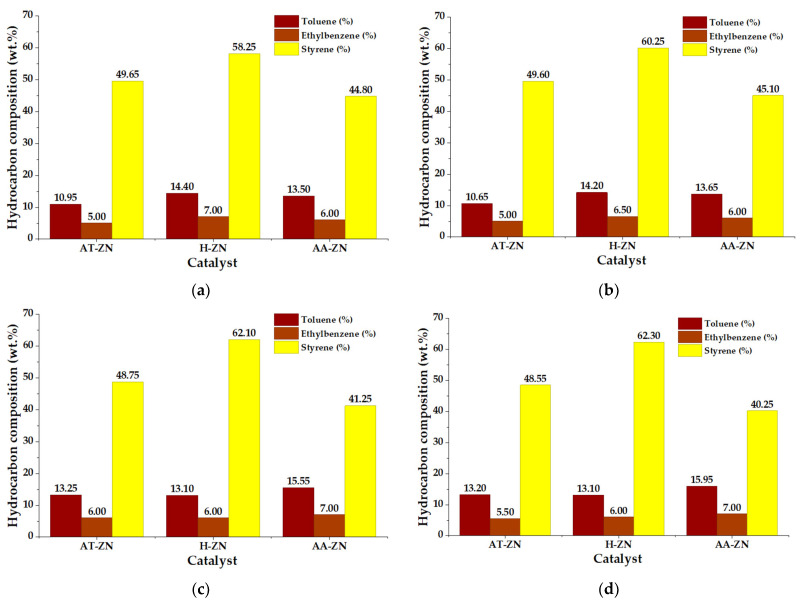
Targeted GC–MS aromatic composition of the pyrolysis oil obtained at 500 °C using AT-ZN, H-ZN, and AA-ZN under equivalent catalyst-loading and heating-rate conditions: (**a**) 5 wt.% catalyst and 10 °C min^−1^; (**b**) 5 wt.% catalyst and 20 °C min^−1^; (**c**) 10 wt.% catalyst and 10 °C min^−1^; and (**d**) 10 wt.% catalyst and 20 °C min^−1^. Toluene, ethylbenzene, and styrene are expressed as relative abundance values within the analyzed aromatic fraction.

Figure 9, Figure 10 and Figure 11 summarize the targeted GC–MS aromatic composition of the pyrolysis oils obtained at 400, 450, and 500 °C, respectively. The discussion is focused on cross-temperature trends and catalyst-dependent behavior, since the compositional changes associated with increasing the heating rate from 10 to 20 °C min^−1^ were comparatively small. Across all evaluated conditions, styrene was the dominant quantified aromatic compound, confirming that PS conversion proceeded mainly through depolymerization pathways rather than complete transformation into lighter alkylbenzenes [14,20,21].

H-ZN consistently showed the highest styrene proportion among the evaluated catalysts. Its high total acidity, dominated by medium- and strong-acid contributions, combined with intermediate textural accessibility, provided a catalytic environment that promoted PS chain cleavage while limiting excessive conversion of primary styrenic intermediates. Although increasing temperature promoted the formation of toluene and ethylbenzene, H-ZN maintained a more styrene-rich profile than AT-ZN and AA-ZN. The results therefore indicate that styrene preservation was associated with an appropriate acidity–accessibility balance rather than with the largest BET surface area [16,19,27].

AT-ZN exhibited an intermediate aromatic profile. Its lower total acidity and moderate textural development were consistent with a less severe catalytic environment, in which styrene remained the dominant quantified product but its proportion was lower than that obtained with H-ZN. The comparatively stable composition across the temperature range agrees with the limited sensitivity of the phase distribution to the operating factors [14,15,29].

AA-ZN showed the lowest styrene proportion and the highest relative contribution of toluene and ethylbenzene, particularly as temperature and catalyst loading increased. Although AA-ZN exhibited the largest BET surface area and pore volume, its substantial medium- and strong-acid contributions and enhanced accessibility favored further transformation of the primary styrenic products. This result confirms that increased surface area alone does not ensure styrene preservation and that an excessively active catalytic environment may promote dealkylation, hydrogen transfer, secondary cracking, and residue-forming reactions [16,19,22,27].

Overall, the GC–MS results indicate that the catalyst modification route had a stronger influence on the aromatic composition than the moderate change in the heating rate. H-ZN was the most favorable material for styrene-rich oil formation, AT-ZN showed an intermediate response, and AA-ZN promoted greater secondary aromatic transformation. The repeated panel-level descriptions were therefore simplified, and the interpretation was reorganized to compare temperature-dependent trends under equivalent heating rates and catalyst loadings. The graphing means, standard deviations, and replicate numbers for toluene, ethylbenzene, and styrene are provided in Table S2. These values describe targeted aromatic composition within the analyzed liquid fraction and should not be interpreted as a complete molecular mass balance of the pyrolysis oil [41,42].

### 3.7. Correlation Analysis of Operating Variables and Aromatic Product Distribution

The Pearson correlation matrices shown in Figure 10 provide a quantitative description of how the operating variables affect the aromatic composition of the pyrolysis oil for each modified zeolite. In all three catalysts, the heating rate exhibited correlations close to zero with toluene, ethylbenzene, and styrene, confirming that, within the studied range, the compositional profile is not significantly governed by the thermal ramp. In contrast, pyrolysis temperature was the most influential variable, showing positive correlations with toluene and ethylbenzene and negative correlations with styrene in the three systems. This pattern indicates that increasing temperature promotes the conversion of primary styrenic intermediates into lighter aromatic by-products, consistent with enhanced secondary cracking, dealkylation, and hydrogen transfer reactions.

For AT-ZN (Figure 10a), temperature showed a moderate positive correlation with toluene and ethylbenzene and a strong negative correlation with styrene, indicating that the aromatic distribution becomes progressively less styrene-selective as thermal severity increases. Catalyst loading displayed a positive relationship with toluene and a weaker positive association with styrene, suggesting that additional AT-ZN slightly modifies the balance between depolymerization and secondary aromatic transformation. However, its influence remains lower than that of temperature. The strong negative correlation between ethylbenzene and styrene further indicates that both species evolve competitively. In contrast, the inverse relationship between toluene and styrene confirms that styrene consumption is accompanied by the formation of secondary monoaromatics [41,42].

For H-ZN (Figure 10b), the correlation pattern reveals a more controlled compositional response. Temperature remained positively correlated with toluene and ethylbenzene and negatively correlated with styrene. However, the inverse relationship between temperature and styrene was weaker than in AT-ZN and AA-ZN, consistent with H-ZN’s superior ability to preserve styrene selectivity. The strong positive correlation between toluene and ethylbenzene suggests that both compounds are formed through related secondary pathways. In contrast, their negative correlations with styrene indicate that they increase at the expense of the desired monomer. Catalyst loading showed only weak correlations with the aromatic products, confirming that, for H-ZN, the composition is governed mainly by catalyst nature and temperature rather than by the amount of catalyst used [41,42].

**Figure 12:**
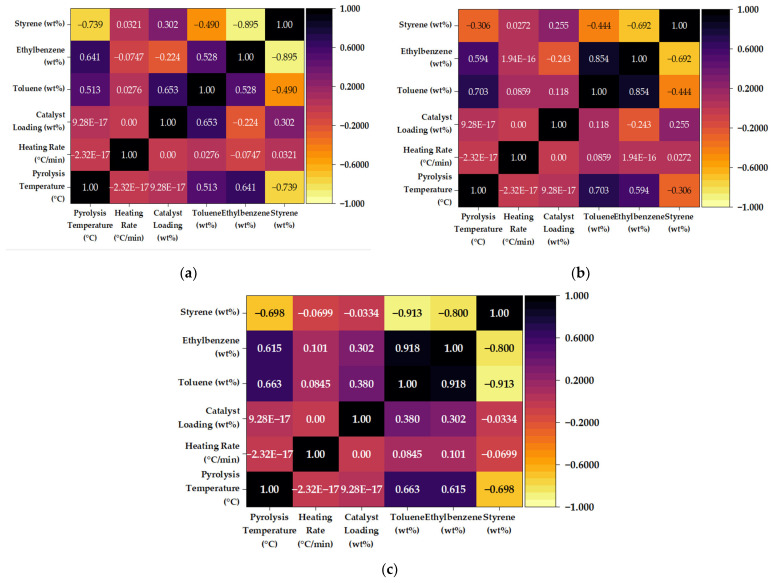
Heatmaps of the Pearson correlation matrices for the variables associated with the aromatic composition of the pyrolysis oil obtained from catalytic pyrolysis of polystyrene using (**a**) AT-ZN, (**b**) H-ZN, and (**c**) AA-ZN.

As shown in Figure 12c, the correlation matrix for AA-ZN reveals the most pronounced shift in aromatic composition. Temperature was strongly and positively correlated with toluene and ethylbenzene and strongly negatively correlated with styrene, indicating that AA-ZN was the catalyst most sensitive to thermally induced secondary reactions. Moreover, the very strong positive correlation between toluene and ethylbenzene, together with the strong negative correlations of both compounds with styrene, indicates a pronounced competitive relationship in which the loss of styrene selectivity was associated with the formation of secondary aromatic products. Catalyst loading exhibited moderate positive correlations with toluene and ethylbenzene but almost no correlation with styrene, suggesting that increasing the amount of AA-ZN primarily promoted secondary conversion rather than monomer preservation.

### 3.8. Mechanistic Interpretation of Product Distribution

**Scheme 1:**
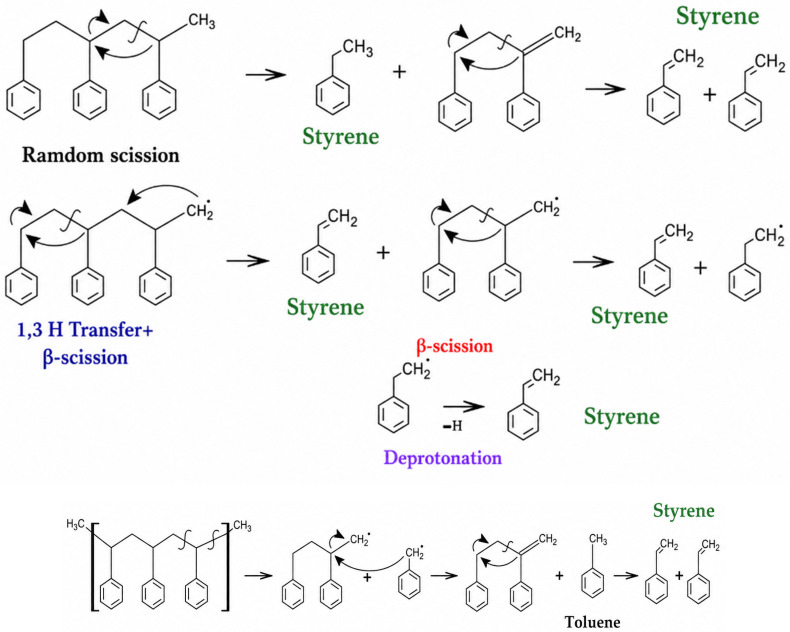
Proposed reaction pathways for the formation of styrene, ethylbenzene, and toluene during the catalytic pyrolysis of polystyrene over modified natural zeolites.

Scheme 1 summarizes the plausible reaction pathways involved in the formation of the main aromatic compounds identified in the liquid fraction during PS pyrolysis, namely styrene, ethylbenzene, and toluene. The mechanism is initiated by random scission of the polymer backbone, which generates macroradical intermediates. These primary radicals may undergo direct depolymerization, releasing styrene, consistent with the well-known tendency of PS to degrade via chain-end scission and unzipping reactions. The predominance of styrene in the GC–MS results indicates that this pathway is the primary route under the conditions evaluated.

A second relevant route involves 1,3-hydrogen transfer followed by β-scission, which also yields styrene from radical intermediates. This pathway is mechanistically important because it explains how the polymer can still yield styrene even after internal rearrangement of the initially formed radicals. In addition, deprotonation of benzylic-type intermediates may further contribute to styrene formation, reinforcing the dominance of this product in the liquid fraction. Altogether, these routes indicate that styrene is primarily associated with controlled depolymerization rather than with extensive secondary cracking [43,44,45].

The formation of ethylbenzene can be explained by hydrogen transfer reactions involving styrene or styrenic radical intermediates. In this context, ethylbenzene may arise from the hydrogenation or stabilization of unsaturated aromatic fragments formed during primary depolymerization. Its presence in a lower proportion than styrene suggests that hydrogen-transfer reactions occur as secondary events, but do not dominate the reaction network under the most selective catalytic conditions.

The formation of toluene is consistent with more extensive secondary transformations of the primary styrenic products. This compound can be associated with cleavage of side-chain fragments, hydrogen redistribution, and further cracking of aromatic intermediates over acid sites. Therefore, a higher toluene fraction indicates a more severe cracking environment, in which the initially formed styrene undergoes further conversion. This interpretation agrees with the catalytic trends observed experimentally, particularly in more acidic systems, where styrene selectivity decreased and the relative abundance of toluene increased [43,44,45].

Recent computational and experimental studies further support the influence of zeolite properties on the product distribution obtained from PS-containing feedstocks. Ma et al. combined reactive molecular dynamics and Monte Carlo simulations to investigate zeolite-assisted PS pyrolysis and demonstrated that zeolite structure, pore accessibility, and adsorption affinity can affect product selectivity. Their results indicated that preferential styrene adsorption may contribute to styrene preservation, whereas strong interfacial interactions between the polymer and the zeolite surface may hinder chain cleavage when polymer segments are excessively stabilized near the interface [46]. From an experimental perspective, Dyer et al. showed that modifying ZSM-5 with different metals altered the yields and compositions of the oil and gas fractions obtained during biomass–PS co-pyrolysis, including the relative distribution of single-ring aromatic compounds [47]. Although their feedstock, catalyst composition, and two-stage reactor configuration differ from those employed in the present study, their findings reinforce that catalyst modification can change both phase distribution and aromatic-product selectivity. Together, these studies support the interpretation that the behavior of the evaluated natural zeolites was governed by the combined influence of pore accessibility, adsorption interactions, and acid-site distribution rather than by a single catalyst descriptor.

### 3.9. Scope and Limitations of the Study

The parent natural zeolite and the freshly modified materials were characterized by XRF, N_2_ adsorption–desorption, NH_3_-TPD, TGA/DTG, and FTIR. These measurements enabled the modification routes to be compared in terms of bulk composition, molar Si/Al ratio, surface area, pore volume, micropore contribution, total acidity, acid-strength distribution, hydration behavior, thermal stability, and aluminosilicate-framework vibrations. Accordingly, AT-ZN, AA-ZN, and H-ZN should be understood as experimentally differentiated catalytic materials rather than solely as preparation-based labels.

Nevertheless, the characterization has specific analytical limits. NH_3_-TPD quantifies total acidity and provides an operational distribution of acid strength but does not independently distinguish Brønsted from Lewis acid sites. In addition, the catalysts recovered after pyrolysis were not characterized. Consequently, changes in pore accessibility, acid-site density, framework organization, carbonaceous deposition, and catalyst deactivation during the reaction could not be directly quantified. Any interpretation involving coke formation or post-reaction structural changes is therefore treated as a plausible explanation rather than as an experimentally demonstrated result.

Another limitation concerns the interpretation of the thermogravimetric profiles. TGA provides information on the apparent mass-loss behavior of PS and PS–zeolite mixtures, but it does not directly identify the chemical nature of the evolved products. Therefore, the observed shift in the degradation profile toward lower temperatures should not be interpreted alone as direct proof of selective depolymerization. Instead, it indicates that the presence of modified zeolites alters the thermal conversion behavior of PS. Direct differentiation between polymer volatilization and chemical depolymerization would require evolved-product analysis, such as TG–FTIR, TG–MS, or Py–GC–MS.

The GC–MS analysis was focused on styrene, toluene, and ethylbenzene as representative aromatic compounds in the liquid fraction. Accordingly, the reported aromatic composition should not be interpreted as a complete molecular mass balance of the pyrolysis oil. The liquid, solid, and gas values describe the global phase distribution, whereas the GC–MS results describe the relative abundance of selected aromatic compounds within the analyzed liquid fraction. In addition, the gaseous fraction was estimated by difference from the liquid and corrected solid fractions; therefore, individual gaseous products were not quantified. This distinction is important because the sum of selected aromatic compounds does not necessarily close the total liquid-product mass balance. These limitations define the current analytical scope of the work. Future studies should extend the characterization to the recovered catalysts and combine broader liquid and gas speciation, direct gas analysis, oxidative TGA, post-reaction N_2_ physisorption, acidity measurements, and catalyst-regeneration tests to establish the evolution of the structure–activity relationship during repeated use [41,42].

The laboratory-scale configuration used in this study also defines the extent to which the results can be compared with larger reactor systems. Previous studies have shown that PS pyrolysis and catalytic cracking can be strongly affected by reactor configuration, residence time, polymer–catalyst contact, vapor-phase transport, and heat-transfer limitations. Therefore, the present results should be interpreted as comparative trends obtained under the evaluated semi-batch laboratory conditions rather than as direct evidence of performance under fixed-bed, fluidized-bed, rotary, pilot-scale, or continuous operation. The comparison with literature systems is useful for contextualizing styrene-rich product formation, but differences in scale, reactor hydrodynamics, and heat- and mass-transfer conditions must be considered when extrapolating the results [17,20,39,42].

Catalyst regeneration and reuse were not experimentally evaluated in the present study. Although oxidative regeneration is commonly used to remove carbonaceous deposits from spent zeolitic catalysts, the regenerated material may undergo changes in acidity, pore accessibility, framework stability, or active-site distribution after repeated pyrolysis–regeneration cycles. Therefore, the possibility of reusing AT-ZN, H-ZN, and AA-ZN after oxidative treatment cannot be confirmed from the current dataset. Future work should include controlled oxidation-regeneration experiments, followed by catalytic reuse tests and post-regeneration characterization, to determine whether the modified natural zeolites retain their activity, selectivity, and structural integrity after multiple cycles [9,10,23,24].

## 4. Conclusions

Modified natural zeolites affected the thermal behavior, phase distribution, and aromatic composition of the liquid fraction obtained from post-consumer PS pyrolysis. Among the evaluated materials, H-ZN provided the most favorable overall response, combining high liquid recovery with low solid-residue formation and the highest styrene proportion among the quantified aromatic compounds. AT-ZN showed intermediate and comparatively stable behavior, whereas AA-ZN generated higher solid fractions and lower styrene preservation, suggesting a greater tendency toward secondary transformation and residue-forming pathways under the evaluated conditions.

Thermogravimetric analysis indicated that the presence of zeolitic materials shifted the apparent PS degradation profile toward lower temperatures. However, this shift should not be interpreted alone as direct proof of selective depolymerization, because TGA mass loss cannot distinguish polymer volatilization from chemical transformation without evolved-product analysis. The product-yield and targeted GC–MS results provide complementary evidence that the reactor experiments generated styrene-rich liquid fractions, but a complete molecular balance of the liquid and gaseous products was outside the scope of the present study.

The physicochemical characterization demonstrated that each modification route produced a distinct catalytic material. Thermal activation preserved the bulk Si/Al ratio but reduced total acidity and hydration-related mass loss. Acid treatment increased the molar Si/Al ratio to 7.02, the BET surface area to 68 m^2^ g^−1^, and the total pore volume to 0.145 cm^3^ g^−1^. Proton exchange generated the highest total acidity, 0.72 mmol NH_3_ g^−1^, with the largest contributions from medium- and strong-acid sites. FTIR and thermal characterization indicated that the principal aluminosilicate framework remained present while the treatments modified the hydration environment, local T–O bonding, and thermal response.

Integration of these properties with the pyrolysis results showed that catalytic performance was not controlled by a single descriptor. AA-ZN produced the largest shift in the PS degradation region and exhibited the highest surface area, but it also promoted greater solid formation and lower styrene preservation. In contrast, H-ZN combined intermediate textural accessibility with the highest acidity and provided the most favorable balance between liquid recovery and styrene preservation. These findings demonstrate that the distribution and accessibility of acid sites, rather than surface area or total acidity considered independently, governed the catalytic response.

The present characterization was limited to the parent and freshly modified zeolites. Post-reaction changes in carbonaceous deposition, pore blockage, acidity, structural stability, and catalyst regeneration were not directly quantified. Future work should therefore focus on the recovered and regenerated catalysts, together with complete liquid and gas speciation, to determine the stability of the measured structure–activity relationships during repeated pyrolysis cycles.

## Figures and Tables

**Table 1:** Bulk oxide composition, loss on ignition, and calculated molar Si/Al ratios of the parent and modified natural zeolites.

Component (wt.%)	ZN	AT-ZN	AA-ZN	H-ZN
SiO_2_	66.0	69.0	78.5	71.5
Al_2_O_3_	12.5	13.0	9.5	12.0
CaO	3.8	4.0	1.1	1.8
Na_2_O	1.8	1.9	0.5	0.6
K_2_O	2.5	2.6	1.3	1.8
MgO	1.2	1.2	0.5	0.7
Fe_2_O_3_	1.5	1.6	1.3	1.4
Other oxides	0.7	0.7	0.6	0.7
Loss on ignition	10.0	6.0	6.7	9.5
Total	100.0	100.0	100.0	100.0

**Table 2:** NH_3_-TPD acidity distribution and textural properties of the parent and modified natural zeolites.

Sample	Weak Acid Sites (mmol g^−1^)	Medium Acid Sites (mmol g^−1^)	Strong Acid Sites (mmol g^−1^)	Total Acidity (mmol NH_3_ g^−1^)
ZN	0.18	0.12	0.05	0.35
AT-ZN	0.12	0.11	0.05	0.28
AA-ZN	0.17	0.23	0.15	0.55
H-ZN	0.16	0.31	0.25	0.72

**Table 3:** Textural properties of the parent and modified natural zeolites determined by N_2_ adsorption–desorption.

Sample	BET Surface Area (m^2^ g^−1^)	Micropore Area (m^2^ g^−1^)	Total Pore Volume (cm^3^ g^−1^)	Micropore Volume (cm^3^ g^−1^)	Mean Pore Diameter (nm)
ZN	24	9	0.075	0.004	12.5
AT-ZN	32	12	0.085	0.006	10.6
AA-ZN	68	25	0.145	0.012	8.5
H-ZN	46	20	0.105	0.010	9.1

**Table 4:** Mass losses of the parent and modified natural zeolites within the selected temperature intervals.

Sample	Weight Loss 30–150 °C, %	Weight Loss 150–350 °C, %	Weight Loss 350–800 °C, %	Total Weight Loss, %
ZN	8.5	3.2	1.8	13.5
AT-ZN	3.2	1.7	1.2	6.1
AA-ZN	3.8	2.2	1.4	7.4
H-ZN	5.0	2.6	1.5	9.1

**Table 5:** FTIR band assignments and qualitative spectral changes in the parent and modified natural zeolites.

Region (cm^−1^)	Assignment	ZN	AT-ZN	AA-ZN	H-ZN
3650–3600	Structural OH groups or Si–OH–Al groups	Weak	Weak	May decrease or broaden	More noticeable after protonation
3500–3200	O–H stretching of adsorbed water	Strong and broad	Clearly decreases	Decreases due to decationization	Intermediate
1650–1620	H–O–H bending vibration	Visible	Lower intensity	Lower intensity	Intermediate
1100–1000	Asymmetric stretching of Si–O–Si/Si–O–Al	Main band, ~1050–1070	Retained	May shift toward higher wavenumbers	Retained, possibly shifted
805–780	Symmetric stretching of the framework	Present	Present	May decrease in intensity	Present
620–590	Ring vibrations of the HEU framework	Present	Slightly lower intensity	May decrease	Present
500–450	T–O bending, where T = Si or Al	Strong	Strong	Moderately modified	Strong

**Table 6:** Analysis of variance (ANOVA) for product yields obtained from PS pyrolysis using the AT-ZN catalyst.

	Degrees of Freedom	Sum of Squares	Mean Square	F-Value	*p*-Value
Temperature	2.0000	25.6225	12.8113	0.6534	0.5378
Heating rate	1.0000	0.2400	0.2400	0.0122	0.9137
Catalyst loading	1.0000	9.6267	9.6267	0.4910	0.4968
Temperature × Heating rate	2.0000	0.0325	0.0163	0.0008	0.9992
Temperature × Catalyst loading	2.0000	26.5358	13.2679	0.6767	0.5267
Heating rate × Catalyst loading	1.0000	0.4267	0.4267	0.0218	0.8852
Temperature × Heating rate × Catalyst loading	2.0000	0.0258	0.0129	0.0007	0.9993
Model	11.0000	62.5100	5.6827	0.2899	0.9755
Error	12.0000	235.2700	19.6058		
Corrected Total	23.0000	297.7800			

**Table 7:** Analysis of variance (ANOVA) for product yields obtained from PS pyrolysis using the AA-ZN catalyst.

	Degrees of Freedom	Sum of Squares	Mean Square	F-Value	*p*-Value
Temperature	2	130.8308	65.4154	987.4025	<0.0001
Heating rate	1	3.0104	3.0104	45.4403	<0.0001
Catalyst loading	1	0.6338	0.6338	9.5660	0.0093
Temperature × Heating rate	2	0.0308	0.0154	0.2327	0.7959
Temperature × Catalyst loading	2	146.0925	73.0463	1102.5849	<0.0001
Heating rate × Catalyst loading	1	0.9204	0.9204	13.8931	0.0029
Temperature × Heating rate × Catalyst loading	2	0.1858	0.0929	1.4025	0.2836
Model	11	281.7046	25.6095	386.5586	<0.0001
Error	12	0.7950	0.0663		
Corrected Total	23	282.4996			

**Table 8:** Analysis of variance (ANOVA) for the effect of operating variables and their interactions on the product-yield response obtained using H-ZN as a catalyst.

Source	Degrees of Freedom	Sum of Squares	Mean Square	F-Value	*p*-Value
Temperature	2	96.5422	48.2711	484.9153	<0.0001
Heating rate	1	0.9235	0.9235	9.2768	0.0111
Catalyst loading	1	16.1635	16.1635	162.3727	<0.0001
Temperature × Heating rate	2	0.4708	0.2354	2.3648	0.1399
Temperature × Catalyst loading	2	114.1060	57.0530	573.1352	<0.0001
Heating rate × Catalyst loading	1	1.3389	1.3389	13.4496	0.0037
Temperature × Heating rate × Catalyst loading	2	0.3201	0.1600	1.6078	0.2440
Model	11	229.8650	20.8968	209.9224	<0.0001
Error	11	1.0950	0.0996		
Corrected Total	22	230.9600			

## Data Availability

The original contributions presented in this study are included in the article/Supplementary Material. Further inquiries can be directed to the corresponding author.

## Supplementary Materials

The following supporting information can be downloaded at: https://www.mdpi.com/article/10.3390/polym18151922/s1.
