# Supplementary material for: Effect of Natural Zeolite Modification Route on the Catalytic Pyrolysis of Post-Consumer Polystyrene Toward Styrene-Rich Liquid Products
Source: Polymers (Basel). 2026 Aug 5;18(15):1922. doi: 10.3390/polym18151922 (PMC13468378; doi:10.3390/polym18151922)
Supplement: Supplementary file 1 [file polymers-18-01922-s001.zip › polymers-4331429-supplementary.pdf]

Supplementary material

Table S1. Product yield distribution from repeated catalytic pyrolysis experiments of polystyrene over modified natural zeolites.

Table S1. Product yields obtained during catalytic pyrolysis of polystyrene using AT-ZN, H-ZN, and AA-ZN catalysts under different temperatures, heating rates, and catalyst loadings. The column Graph corresponds to the mean value used in the main figures, while SD represents the sample standard deviation calculated from the replicate experiments. Liquid, solid, and gas fractions are expressed as wt.%.

Catalyst	T (°C)	Heating rate (°C min^{-1})	Catalyst loading (wt.%)	n	Liquid Graph (mean, wt.%)	Liquid SD	Solid Graph (mean, wt.%)	Solid SD	Gas Graph (mean, wt.%)	Gas SD
**AT-ZN**	400	10	5	3	66.48	0.08	12.17	0.21	21.43	0.12
**AT-ZN**	400	20	5	3	65.87	0.06	12.13	0.12	21.87	0.12
**AT-ZN**	400	10	10	3	70.03	5.00	15.87	2.10	14.13	7.10
**AT-ZN**	400	20	10	3	70.07	5.50	15.88	1.65	14.02	7.15
**AT-ZN**	450	10	5	3	66.73	0.06	12.07	0.12	21.33	0.21
**AT-ZN**	450	20	5	3	66.37	0.40	11.33	0.21	22.37	0.40
**AT-ZN**	450	10	10	3	67.53	2.50	13.97	2.00	18.53	4.50
**AT-ZN**	450	20	10	3	67.67	2.00	14.23	2.20	18.07	4.20
**AT-ZN**	500	10	5	3	66.38	0.08	12.12	0.16	21.43	0.12
**AT-ZN**	500	20	5	3	65.97	0.21	12.03	0.12	22.02	0.08
**AT-ZN**	500	10	10	3	65.03	5.00	12.87	2.90	22.13	7.90
**AT-ZN**	500	20	10	3	64.87	5.20	12.88	3.05	22.22	8.25
**H-ZN**	400	10	5	3	86.28	0.26	2.72	0.26	11.03	0.06
**H-ZN**	400	20	5	3	85.62	0.16	2.93	0.31	11.42	0.16
**H-ZN**	400	10	10	3	78.38	0.16	3.62	0.16	17.98	0.08
**H-ZN**	400	20	10	3	78.12	0.16	3.98	0.16	17.87	0.31
**H-ZN**	450	10	5	3	79.78	0.26	2.02	0.08	18.20	0.70
**H-ZN**	450	20	5	3	78.47	0.31	2.28	0.08	19.25	0.26
**H-ZN**	450	10	10	3	78.63	0.12	3.32	0.16	18.03	0.06
**H-ZN**	450	20	10	3	78.27	0.31	3.33	0.21	18.27	0.50
**H-ZN**	500	10	5	3	75.88	0.35	2.62	0.16	21.53	0.50
**H-ZN**	500	20	5	3	76.97	2.00	3.23	0.21	19.77	2.20
**H-ZN**	500	10	10	3	78.33	0.21	3.57	0.12	18.13	0.12
**H-ZN**	500	20	10	2	79.05	0.07	3.55	0.07	17.35	0.07
**AA-ZN**	400	10	5	3	61.58	0.08	23.07	0.12	15.43	0.12
**AA-ZN**	400	20	5	3	60.92	0.26	23.53	0.12	15.47	0.21
**AA-ZN**	400	10	10	3	68.13	0.12	19.87	0.12	12.03	0.06
**AA-ZN**	400	20	10	3	67.07	0.12	20.13	0.12	12.77	0.21
**AA-ZN**	450	10	5	3	61.73	0.21	23.02	0.08	15.23	0.31
**AA-ZN**	450	20	5	3	61.32	0.16	22.63	0.21	16.02	0.08
**AA-ZN**	450	10	10	3	60.18	0.16	24.47	0.12	15.38	0.08
**AA-ZN**	450	20	10	3	59.07	0.12	24.68	0.16	16.22	0.26
**AA-ZN**	500	10	5	3	61.58	0.08	22.92	0.08	15.53	0.06
**AA-ZN**	500	20	5	3	61.52	0.26	22.73	0.06	15.67	0.31
**AA-ZN**	500	10	10	3	56.78	0.26	24.97	0.06	18.28	0.26
**AA-ZN**	500	20	10	3	55.47	0.31	25.28	0.08	19.22	0.26

Table S2. GC–MS aromatic composition of pyrolysis oils obtained from repeated catalytic pyrolysis experiments of polystyrene.

Table S2. Relative abundance of toluene, ethylbenzene, and styrene in the pyrolysis oil obtained from catalytic pyrolysis of polystyrene over AT-ZN, H-ZN, and AA-ZN. The column Graph corresponds to the mean value used in the main figures, while SD represents the sample standard deviation calculated from the replicate experiments. Compound abundances are expressed as relative percentage values determined by GC–MS.

Catalyst	T (°C)	Heating rate (°C min^{-1})	Catalyst loading (wt.%)	n	Toluene Graph (mean, %)	Toluene SD	Ethylbenzene Graph (mean, %)	Ethylbenzene SD	Styrene Graph (mean, %)	Styrene SD
**AT-ZN**	400	10	5	3	10.88	0.16	4.97	0.06	49.83	0.21
**AT-ZN**	400	20	5	3	10.82	0.08	5.03	0.06	50.47	0.31
**AT-ZN**	400	10	10	3	10.78	0.75	3.97	1.00	52.53	3.70
**AT-ZN**	400	20	10	3	11.02	0.75	4.03	1.00	53.12	3.65
**AT-ZN**	450	10	5	3	11.08	0.08	4.97	0.06	49.93	0.12
**AT-ZN**	450	20	5	3	11.07	0.06	5.53	0.50	49.67	0.31
**AT-ZN**	450	10	10	3	12.03	3.00	4.97	1.00	50.68	1.85
**AT-ZN**	450	20	10	3	12.27	2.90	4.53	1.50	50.22	1.85
**AT-ZN**	500	10	5	3	10.98	0.08	4.97	0.06	49.68	0.35
**AT-ZN**	500	20	5	3	10.62	0.16	5.03	0.06	49.57	0.12
**AT-ZN**	500	10	10	3	13.28	0.75	5.97	1.00	48.78	3.75
**AT-ZN**	500	20	10	3	13.17	1.10	5.53	0.50	48.52	4.35
**H-ZN**	400	10	5	3	11.53	0.31	5.47	0.50	61.78	0.26
**H-ZN**	400	20	5	3	11.82	0.26	6.03	0.06	60.67	0.21
**H-ZN**	400	10	10	3	13.03	0.06	5.97	0.06	62.18	0.16
**H-ZN**	400	20	10	3	13.07	0.06	6.03	0.06	62.87	0.21
**H-ZN**	450	10	5	3	12.63	0.60	5.97	0.06	64.63	0.40
**H-ZN**	450	20	5	3	12.97	0.50	6.03	0.06	63.32	0.16
**H-ZN**	450	10	10	3	13.13	0.12	5.97	0.06	62.18	0.16
**H-ZN**	450	20	10	3	13.22	0.08	6.03	0.06	61.97	0.21
**H-ZN**	500	10	5	3	14.43	0.12	6.97	0.06	58.28	0.75
**H-ZN**	500	20	5	3	14.17	0.80	6.53	0.50	60.22	2.25
**H-ZN**	500	10	10	3	13.13	0.12	5.97	0.06	62.13	0.12
**H-ZN**	500	20	10	2	13.05	0.07	6.05	0.07	62.25	0.07
**AA-ZN**	400	10	5	3	13.63	0.12	5.97	0.06	45.08	0.08
**AA-ZN**	400	20	5	3	13.32	0.08	6.03	0.06	45.17	0.21
**AA-ZN**	400	10	10	3	12.48	0.08	5.47	0.50	50.28	0.26
**AA-ZN**	400	20	10	3	12.82	0.08	6.03	0.06	49.62	0.16
**AA-ZN**	450	10	5	3	13.48	0.08	5.97	0.06	44.88	0.16
**AA-ZN**	450	20	5	3	13.27	0.12	6.03	0.06	44.67	0.12
**AA-ZN**	450	10	10	3	14.08	0.08	5.97	0.06	44.13	0.12
**AA-ZN**	450	20	10	3	14.47	0.12	6.03	0.06	43.07	0.12
**AA-ZN**	500	10	5	3	13.53	0.06	5.97	0.06	44.83	0.21
**AA-ZN**	500	20	5	3	13.62	0.08	6.03	0.06	45.07	0.21
**AA-ZN**	500	10	10	3	15.58	0.08	6.97	0.06	41.28	0.26
**AA-ZN**	500	20	10	3	15.92	0.08	7.03	0.06	40.22	0.26
